# Supplementary material for: Structure and transport mechanism of P5B-ATPases
Source: Nat Commun. 2021 Jun 25;12:3973. doi: 10.1038/s41467-021-24148-y (PMC8233418; doi:10.1038/s41467-021-24148-y)
Supplement: Supplementary file 1 — Supplementary Information [file 41467_2021_24148_MOESM1_ESM.pdf]

Supplementary Information:

Structure and transport mechanism of P5B-ATPases

Ping Li[1*], Kaituo Wang[2*], Nina Salustros[2], Christina Grønberg[2] & Pontus Gourdon[1,2,†]

[1] Department of Experimental Medical Science, Lund University, Sölvegatan 19, SE-221 84 Lund, Sweden

[2] Department of Biomedical Sciences, Copenhagen University, Maersk Tower 7-9, Nørre Allé 14, DK-2200 Copenhagen N, Denmark

[*] Contributed equally

[†] Correspondence should be addressed to: P. G. (pontus.gourdon@med.lu.se)

Supplementary Table 1. Cryo-EM data collection, data processing and model building statistics.

	E2P^{inhib}	E2P*	E2.P$_i^{AlF/SPM}$	E2.P$_i^{SPM}$
	**PDB 7OP8, EMD-13014**	**PDB 7OP5, EMD-13013**	**PDB 7OP1, EMD-13011**	**PDB 7OP3, EMD-13012**
**Data collection**				
EM equipment	FEI Titan Krios	FEI Titan Krios	FEI Titan Krios	FEI Titan Krios
Voltage (kV)	300	300	300	300
Detector	Falcon 3	Gatan K3	Gatan K3	Falcon 3
Data collection mode	counting	Super resolution	counting	counting
Pixle size (Å)	0.83	1.09	0.86	0.83
Energy filter	/	20	/	/
Electron dose (e^-/Å^2)	40	50	50	40
Defocus range (mm)	-1.2 ~ -2.6	-1.2 ~ -2.8	-1.2 ~ -2.6	-1.2 ~ -2.6
**Data processing**				
Software	cryosparc	cryosparc	cryosparc	cryosparc
Number of final used particles	51552	141028	59345	51935
Symmetry	C1	C1	C1	C1
Map resolution (Å)	3.5	3.7	3.4	3.5
**Model refinement statistics**				
Total built residues	1118	1076	1098	1100
Model-map-fit CC	0.77	0.79	0.86	0.81
**R.m.s.d.**				
bonds (Å)	0.004	0.004	0.004	0.003
angles (°)	0.846	0.692	0.600	0.776
**Molprobity statistics**				
Molprobity score	1.73	1.86	1.70	1.65
**Ramachandran plot**				
Favored (%)	94.36	93.75	95.29	95.22
Allowed (%)	5.64	6.25	4.71	4.78
Rotamer outliers (%)	0	0	0	0
Clash score	6.24	8.16	6.69	5.89
Average B-factor (Å^2)	69.03	55.90	61.08	87.27

Supplementary Figure 1a.

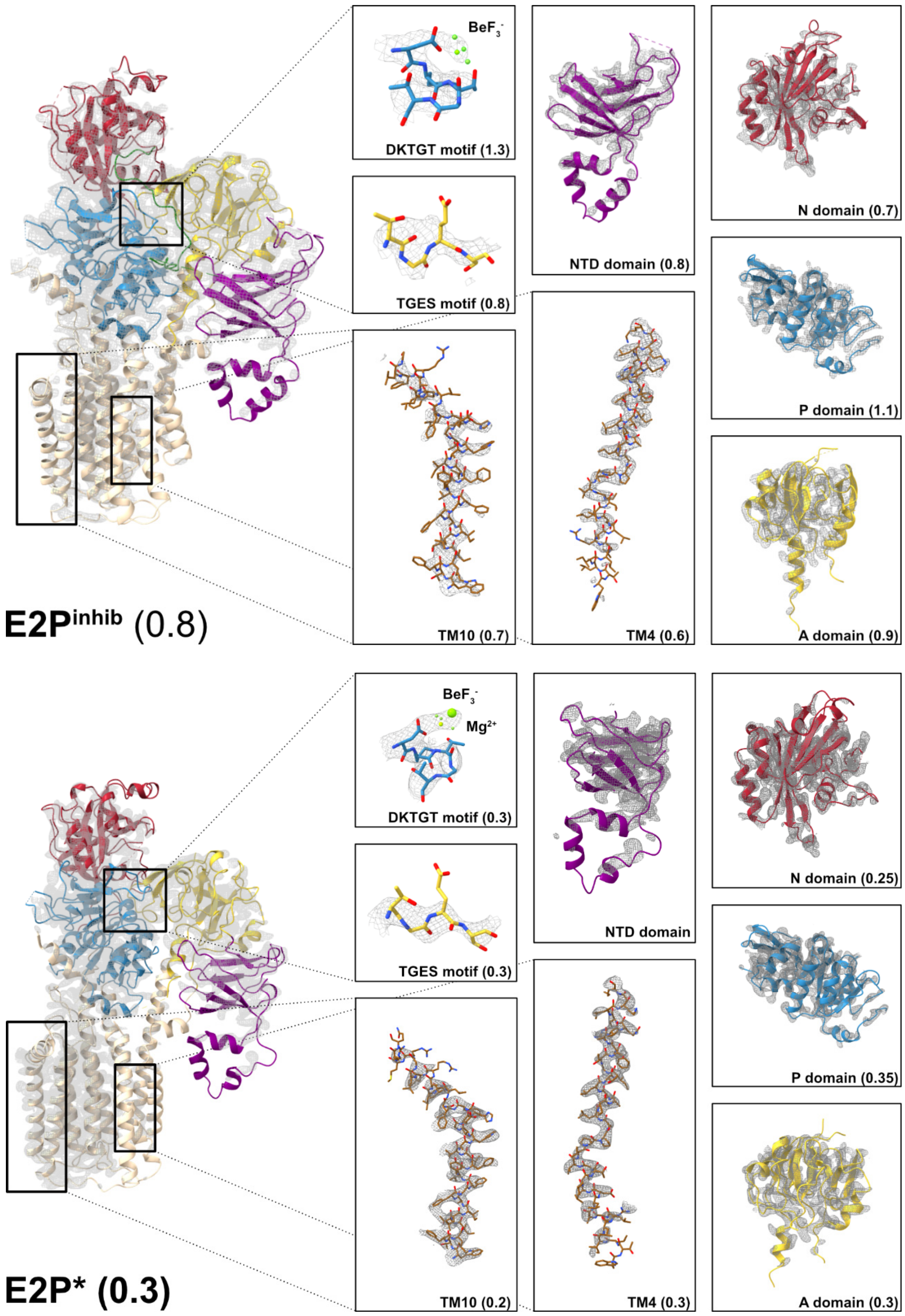

Supplementary Figure 1b. Map quality of E2P^{inhib} and E2P* states. Contour levels are indicated in brackets.

Supplementary Figure 1b.

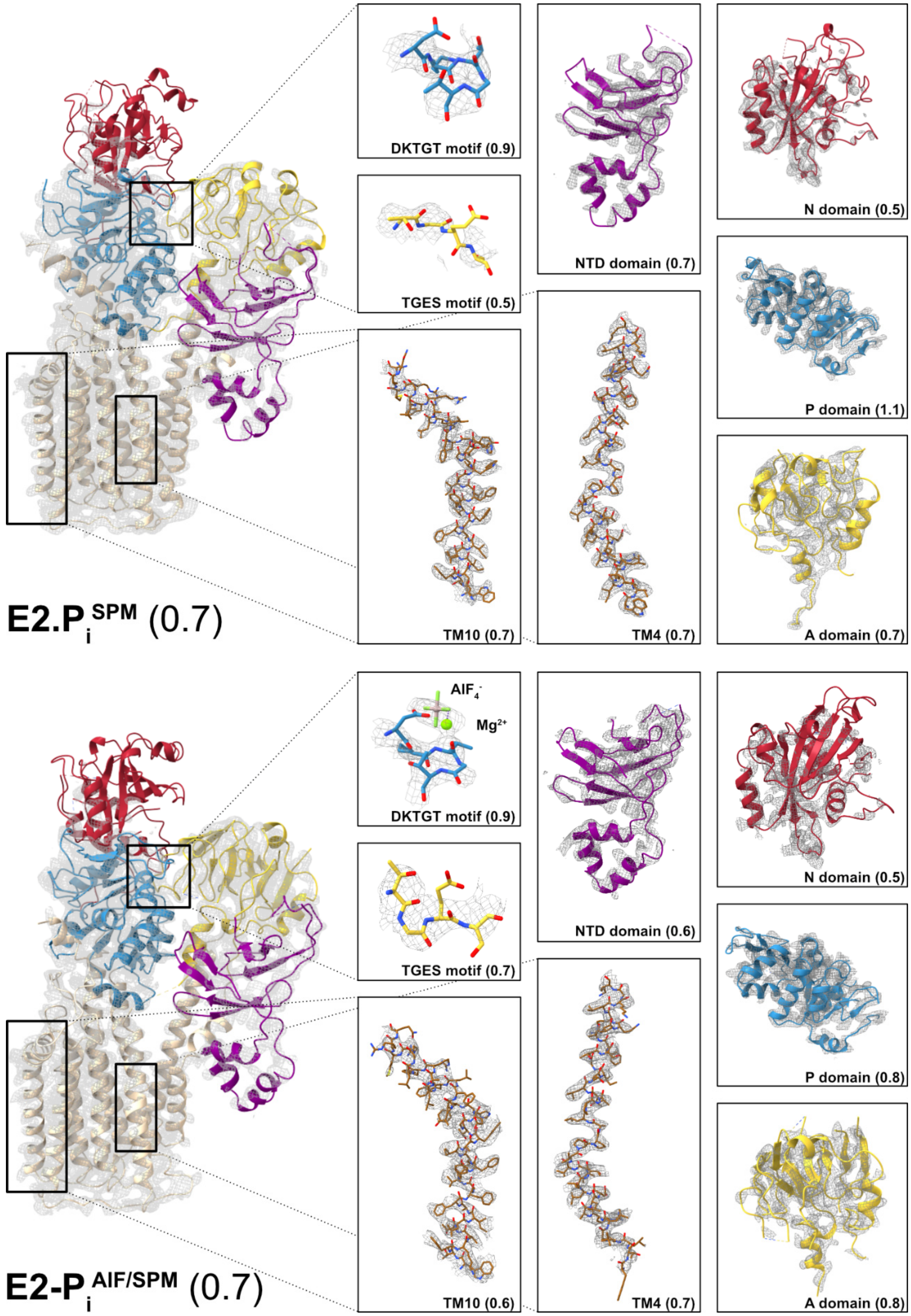

Supplementary Figure 1b. Map quality of E2P^{inhib} and E2P* states. Contour levels are indicated in brackets.

Supplementary Figure 2.

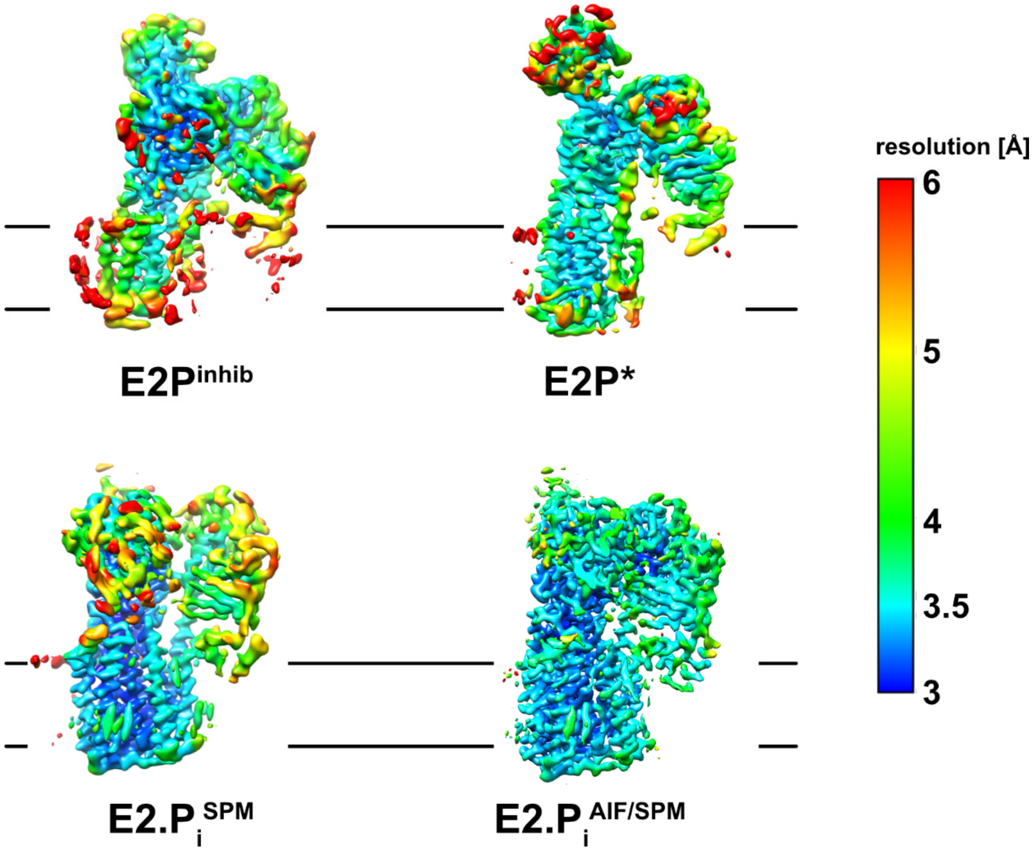

Supplementary Figure 2. Local resolution of the determined Ypk9 cryo-EM maps.

Supplementary Figure 3.

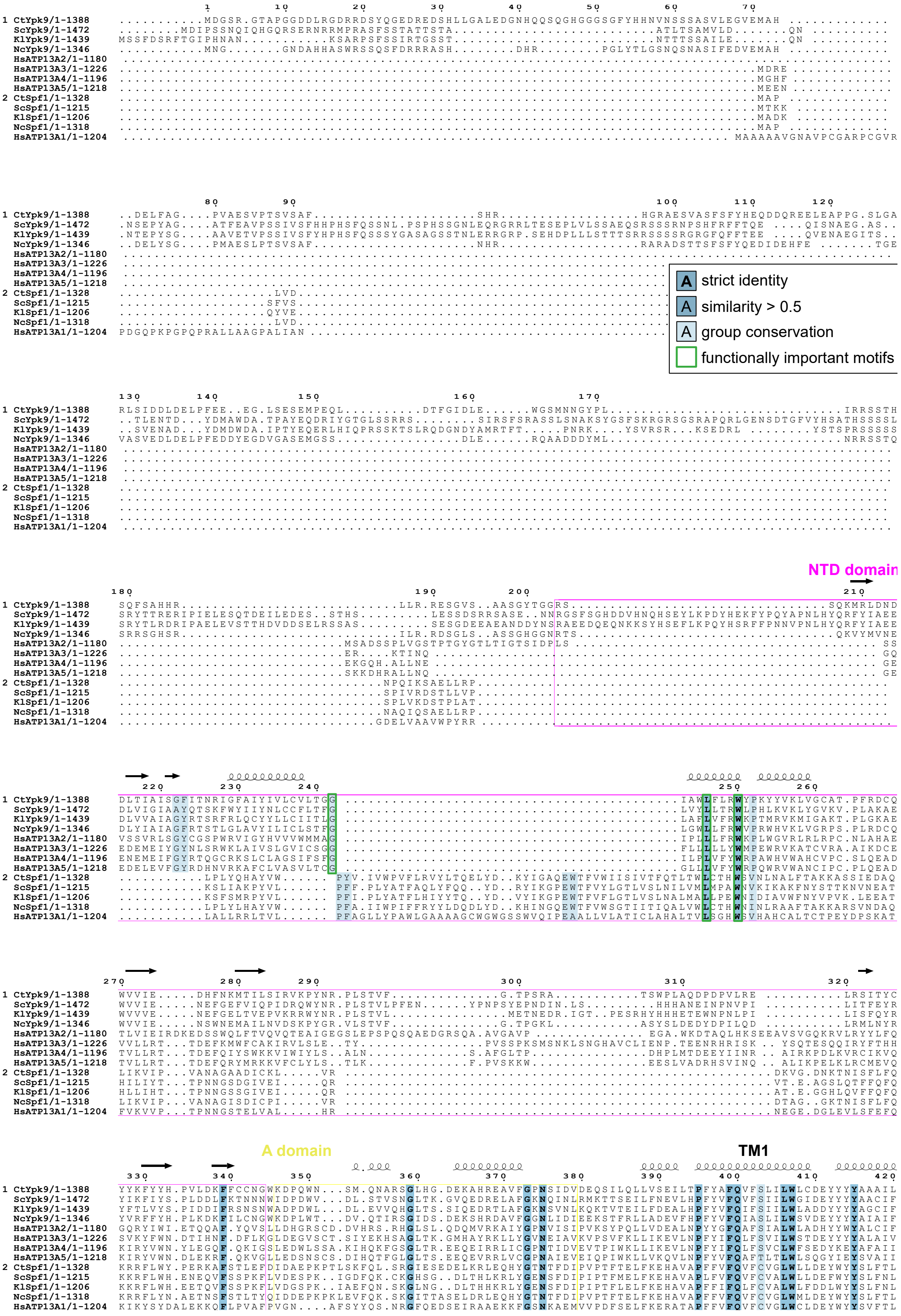

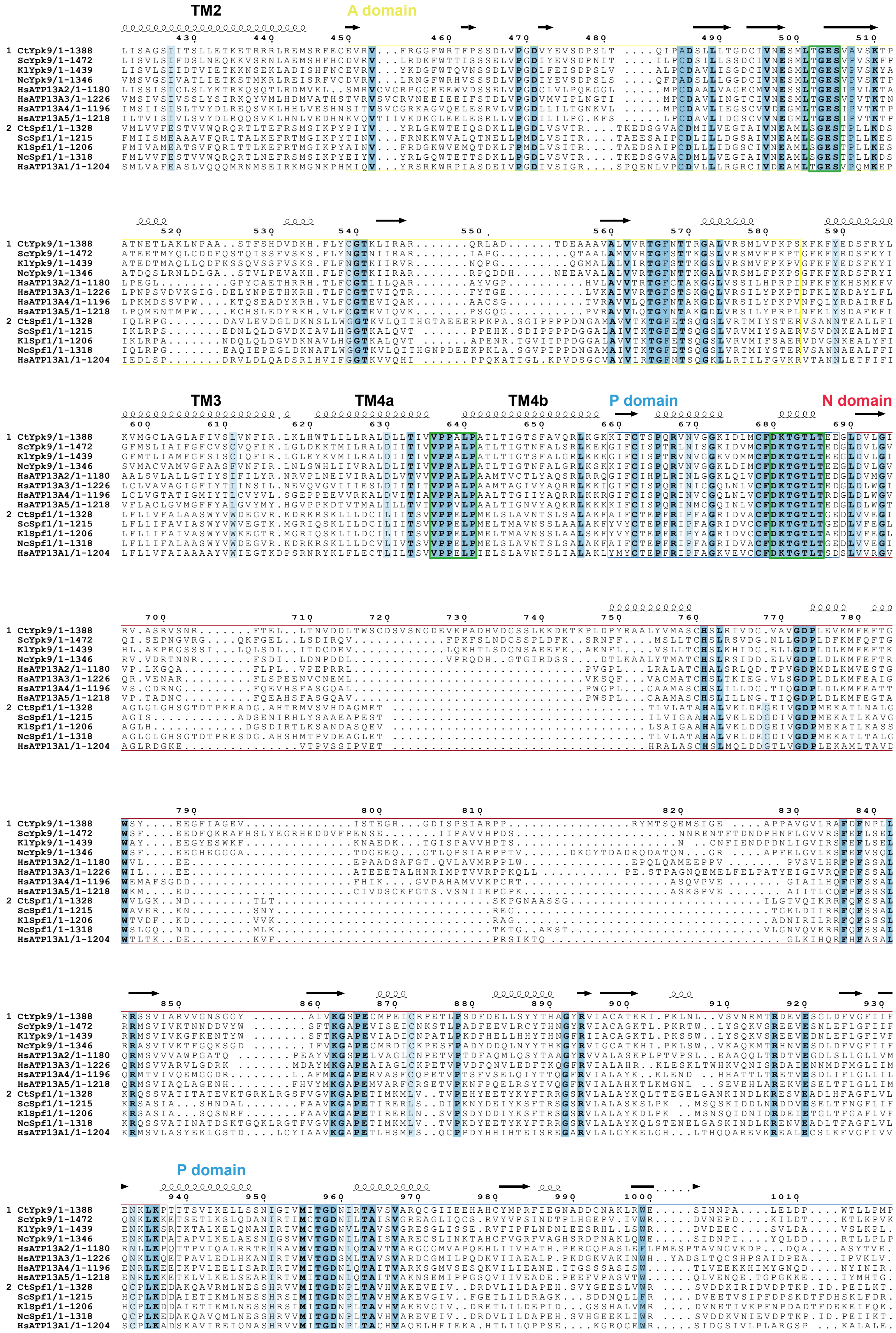
TM2
A domain
TM3
TM4a
TM4b
P domain
N domain
P domain

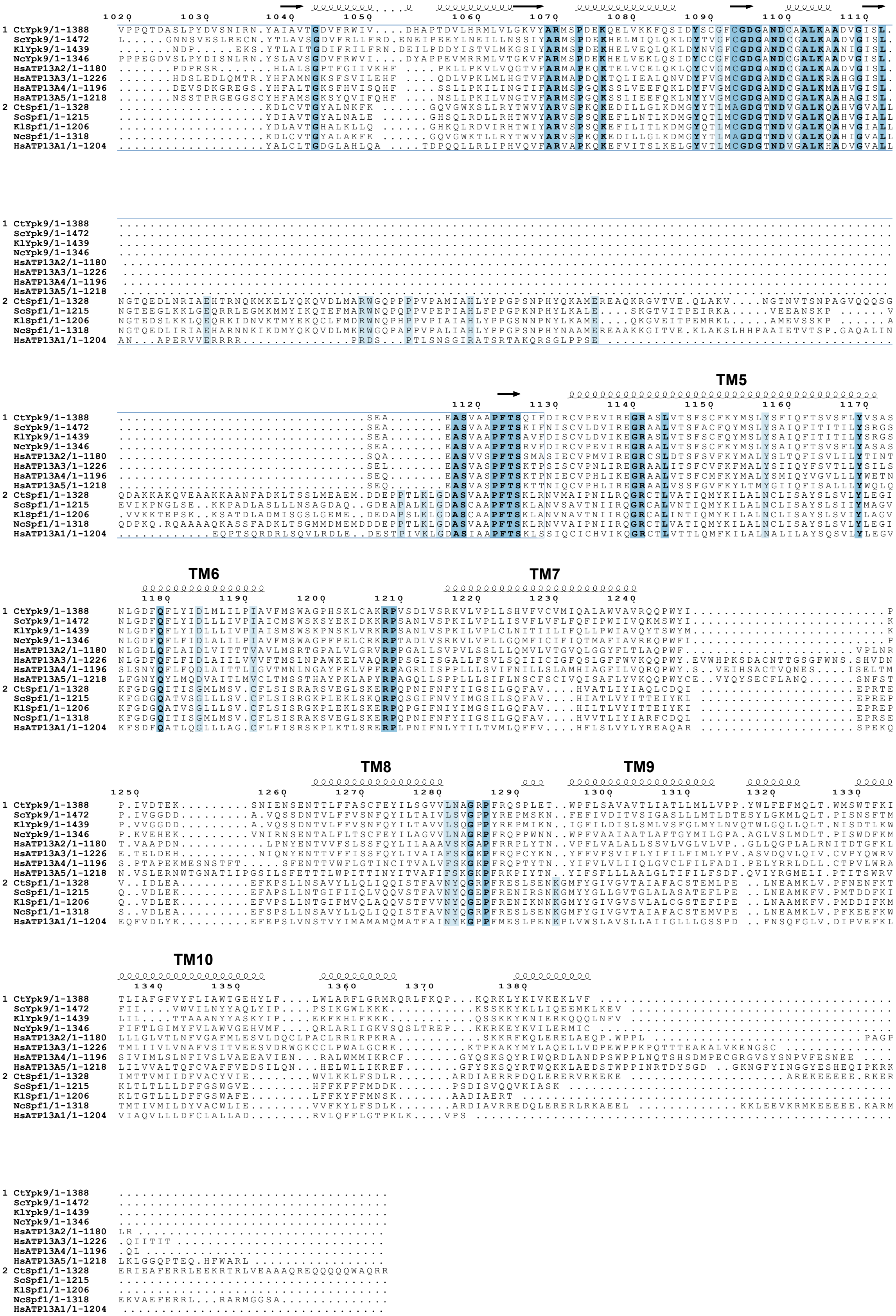

TM5
TM6
TM7
TM8
TM9
TM10
1 CtYpk9/1-1388
ScYpk9/1-1472
KlYpk9/1-1439
NcYpk9/1-1346
HsATP13A2/1-1180
HsATP13A3/1-1226
HsATP13A4/1-1196
HsATP13A5/1-1218
2 CtSpf1/1-1328
ScSpf1/1-1215
KlSpf1/1-1206
NcSpf1/1-1318
HsATP13A1/1-1204

Supplementary Figure 3. Structure-based multiple sequence alignment of selected P5B- (group 1) and P5A- (group 2) ATPases. The following sequences are included in the alignment (Uniprot ID in brackets): CtYpk9 (G0S7G9), ScYpk9 (Q12697), KlYpk9 (A0A5P2UE38), NcYpk9 (A7UWZ1), HsATP13A2-5 (Q9NQ11, Q9H7F0, Q4VNC1, Q4VNC0), CtSpf1 (G0S4Z4), ScSpf1 (P39986), KlYpk9 (Q6CM81), NcYpk9 (Q7S1D7), HsATP13A1 (Q9HD20). Alignments were performed using Clustal Omega [44] and visualized using ESPript 3.0 [45]. Similarity scores were calculated using the BLOSUM62 matrix. Manual modifications were conducted in the N-terminal domain based on the determined structures.

Supplementary Figure 4.

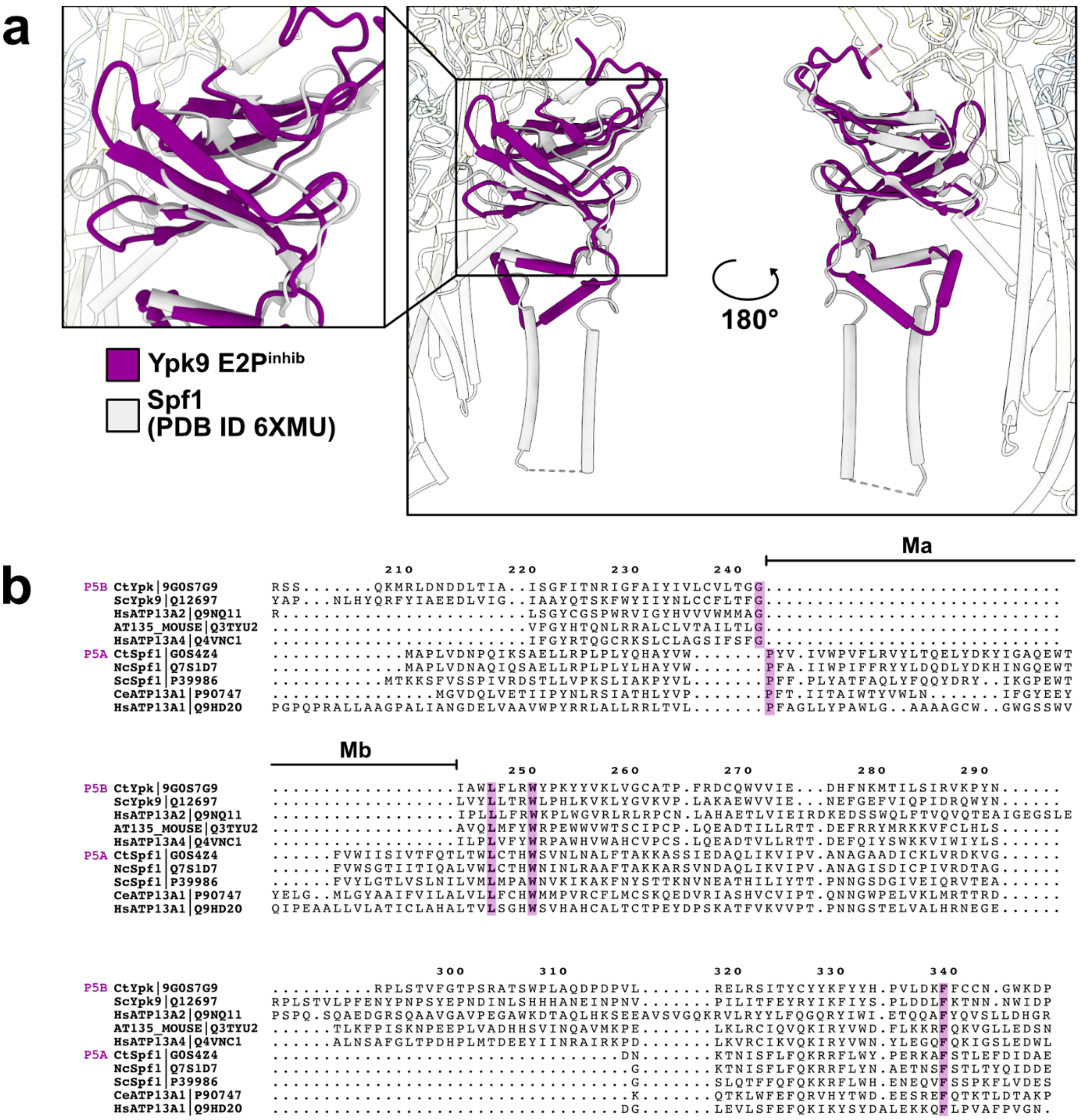

Supplementary Figure 4. Structural alignments of the NTD-domains of Ypk9 in the E2P^{inhib} state to the P5A-ATPase Spf1. a The NTD of Spf1 has two transmembrane helices Ma and Mb, while Ypk9 instead harbors a membrane-dipping loop. The soluble parts superpose well, and both structures feature a seven-stranded β-barrel fold. The NTD domain harbors residues R204-G344 and is thus preceded by approximately 200 residues in Ypk9, of which D74-G96 are modelled. **b** Structure-based sequence alignment of the NTD of select P5A- and P5B-ATPases. Identical residues are highlighted, and Uniprot IDs are indicated. We note the length of the N-terminus prior to the NTD varies among P5-ATPases (see also Supplementary Figures 3 and 11).

Supplementary Figure 5.

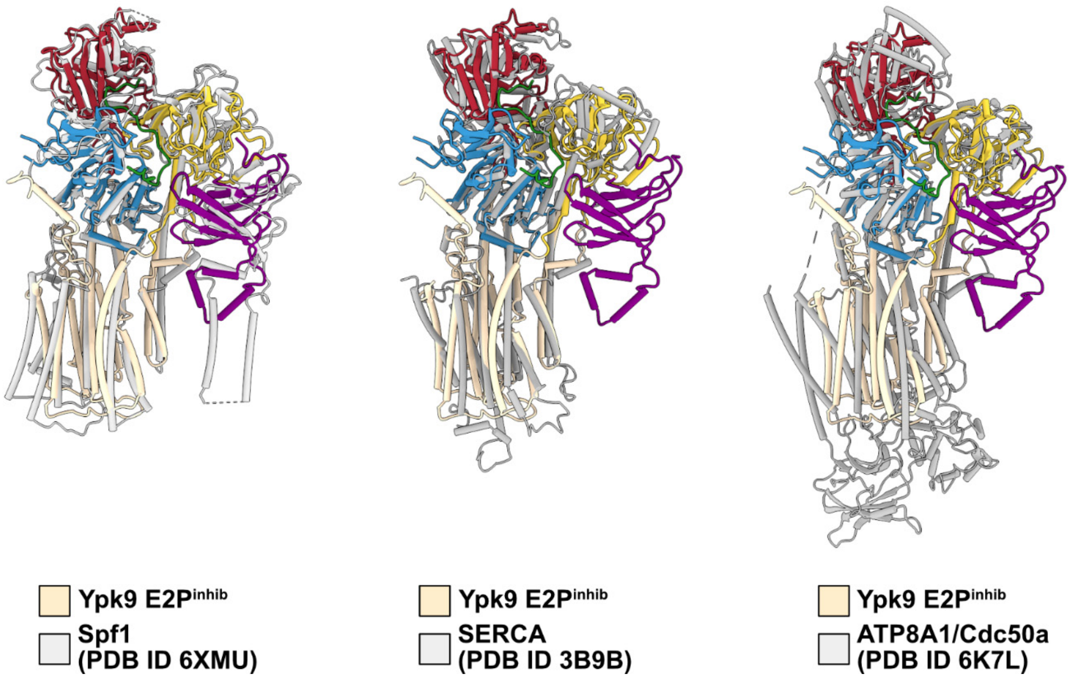

Supplementary Figure 5. Structural alignments of Ypk9 in the E2P^{inhib} state to the P5A-ATPase Spf1, the sarco/endoplasmic reticulum Ca^{2+}-ATPase (SERCA) and the P4-ATPase ATP8A1/Cdc50a.

Supplementary Table 2. Structural alignments of the determined Ypk9 structures to the soluble domains of a the sarco/endoplasmic reticulum Ca^{2+}-ATPase (SERCA), and b the P4-ATPase ATP8A1-Cdc50a. RMSD values are indicated. Our data resemble two different conformations, i.e. E2P ($E2P^{inhib}$ and E2P*) and $E2.P_i$ ($E2.P_i^{SPM}$ and $E2.P_i^{AlF/SPM}$) as highlighted in grey.

a

**SERCA state**	PDB ID	7OP8	7OP5	7OP3	7OP1
		**$E2P^{inhib}$**	**E2P***	**$E2.P_i^{SPM}$**	**$E2.P_i^{AlF/SPM}$**
E1	4H1W	7.76	8.18	7.22	8.16
[Ca]2 E1	2C9M	4.06	3.83	5.46	5.07
[Ca]2 E1	1SU4	5.46	5.18	7.35	7.82
[Ca]2 E1×ATP	3N8G	9.32	8.87	8.08	8.26
[Ca]2 E1×ATP					
[Ca]2 E1P-ADP	1T5S	10.69	10.88	8.79	12.53
[Ca]2 E1-ADP:AlF4	1T5T	9.56	9.88	8.38	10.56
[Ca]2 E1P:ADP	3BA6	9.97	9.96	8.54	12.50
E2P	3B9B	2.67	2.71	3.94	4.08
E2-P	3N5K	4.26	3.94	2.72	2.24
E2:Pi	3FGO	4.01	4.31	2.73	2.64
E2	3NAL	5.97	6.34	4.93	4.46

b

**ATP8A1-Cdc50a state**	PDB ID	7OP8	7OP5	7OP3	7OP1
		**$E2P^{inhib}$**	**E2P***	**$E2.P_i^{SPM}$**	**$E2.P_i^{AlF/SPM}$**
E1	6K7G	7.44	7.71	8.57	9.65
E1	6K7H	6.49	6.21	7.07	6.33
E1 ATP	6K7I	7.08	7.09	6.83	5.95
E1 ATP	6K7J	8.97	9.01	9.72	6.93
E1P	6K7N	6.68	6.78	8.14	6.28
E1 ADP Pi	6K7K	9.15	9.35	10.28	7.95
E2P	6K7L	4.51	4.50	6.00	5.48
E2Pi - PL	6K7M	5.17	5.26	4.18	4.99

Supplementary Figure 6.

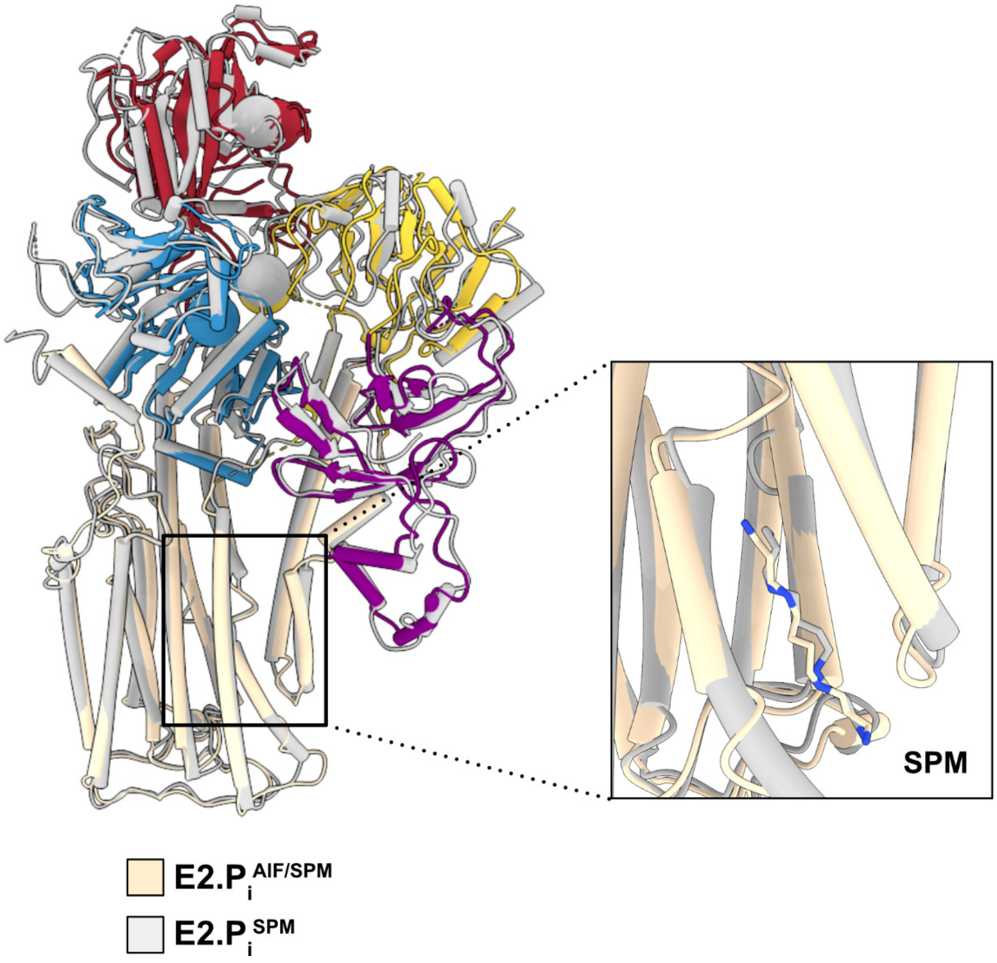

Supplementary Figure 6. Structural alignments of the determined E2.P$_i$ states of Ypk9. Close-view illustrates the bound SPM molecule.

Supplementary Figure 7.

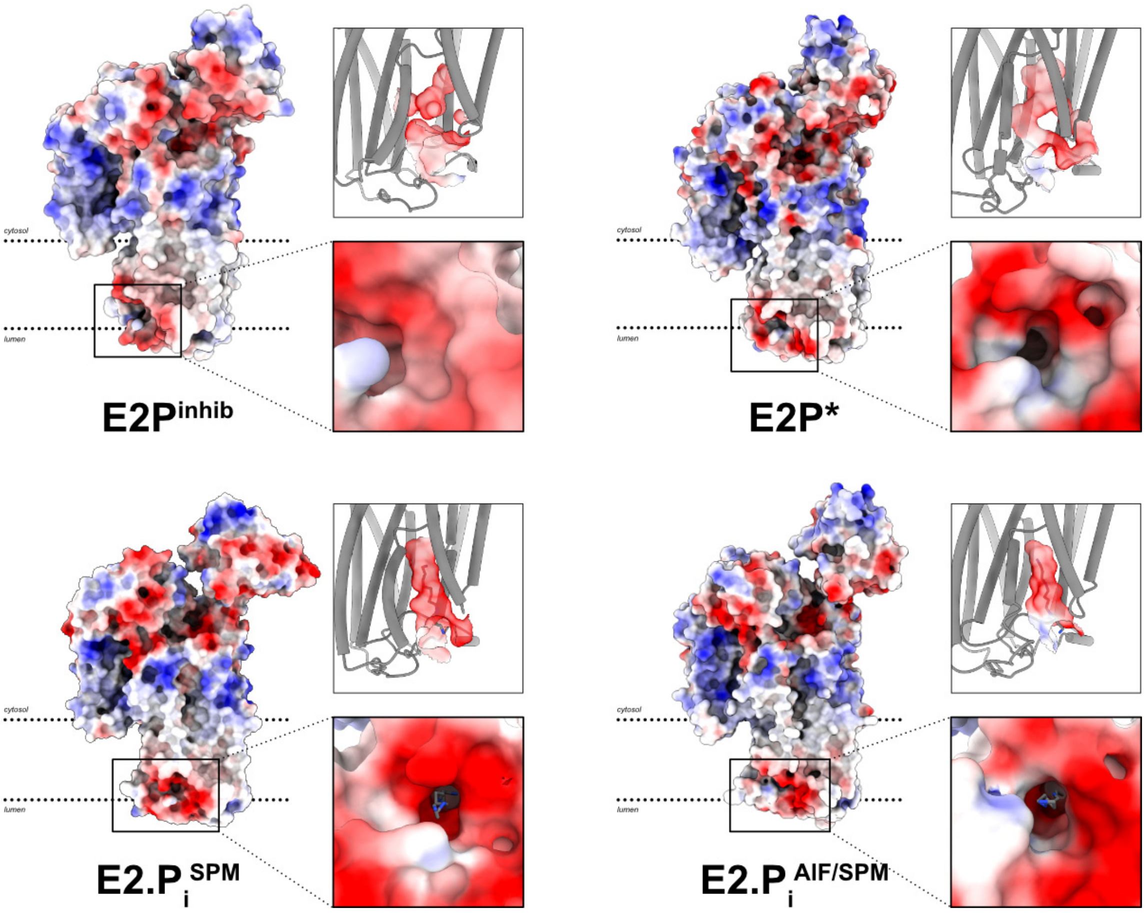

Supplementary Figure 7. Electrostatic surface representation of the determined Ypk9 structures. All structures have an electronegative luminal surface in agreement with SPM uptake. Close-views show SPM binding cavity as seen from the luminal side, and side views of the inner solvent-accessible surface around the SPM binding site. The SPM pocket is highly electronegative, and clearly formed in $E2.P_i^{SPM}$ and $E2P_i^{AlF/SPM}$ states.

Supplementary Figure 8.

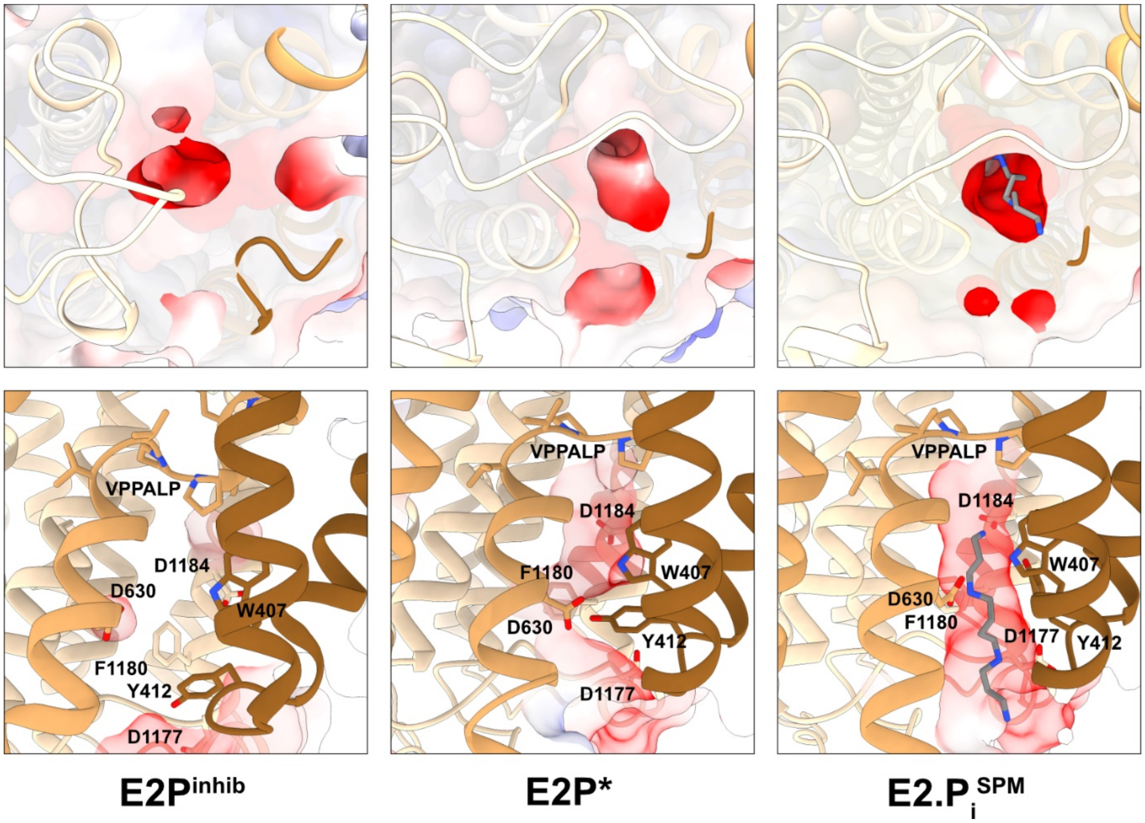

Supplementary Figure 8. Architecture of the SPM binding pocket. Slices through the model surface are presented. Top: View from lumen. Bottom: View from side, with conserved residues shown as sticks. No SPM density is visible in $E2P^{inhib}$, and the pocket is not yet formed. E2P* represents a transition state, and in the $E2.P_i^{SPM}$ state, SPM accommodates the cavity well. While not supported by our data, SPM binding to the E2P states cannot be excluded.

Supplementary Figure 9.

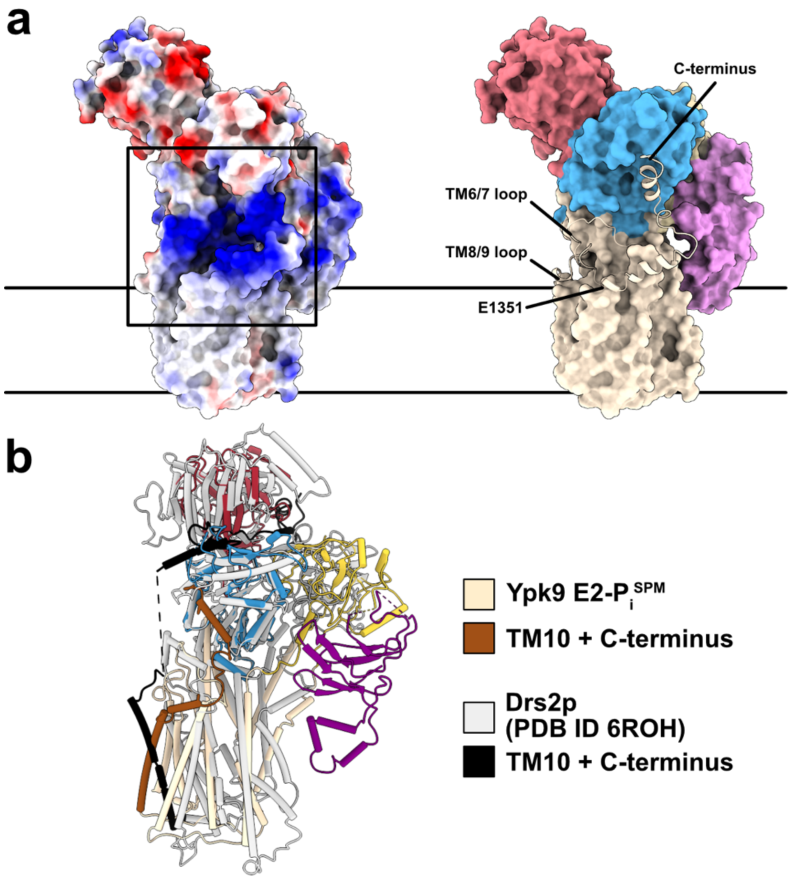

Supplementary Figure 9. The C-terminal region. a Electrostatic surface representation of the C-terminal region reveals a highly electropositive area, mainly formed by the soluble part of TM10 and the P-domain. This region therefore forms a possible interaction site for regulatory lipids. **b** Alignment of Ypk9 to the P4-ATPase Drsp2 in the auto-inhibited state, revealing different orientations of TM10 and the connected C-terminus.

Supplementary Figure 10.

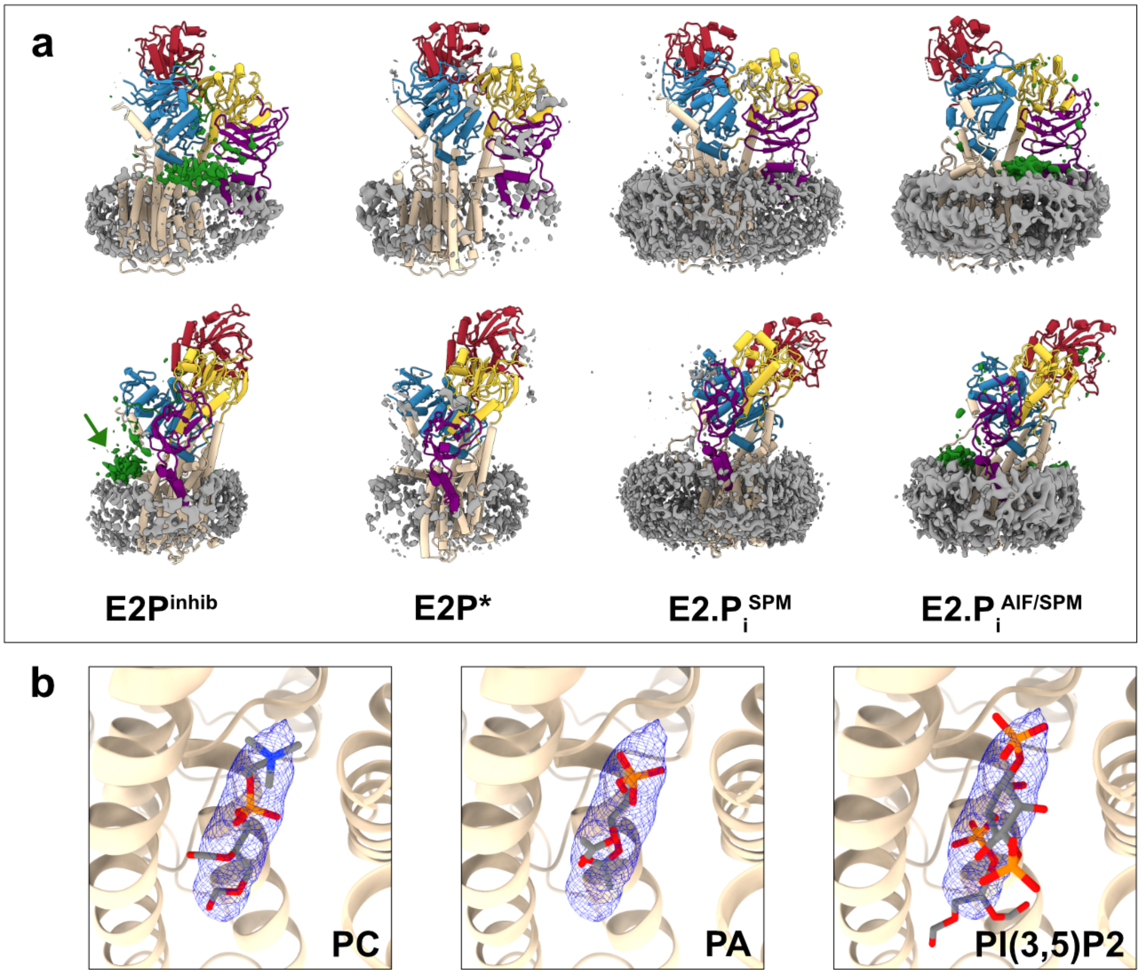

Supplementary Figure 10. Unassigned density. a The EM density around final models was subtracted from the experimental EM maps, and the remaining densities are shown at similar contour levels. In the $E2P^{inhib}$ and $E2.P_i^{AlF/SPM}$ data, additional density is located on the cytosolic side of the membrane (green), likely corresponding to N-terminal residues, bridging the NTD and the autoinhibitory loop. **b** Fit of PC, PA and PI(3,5)P2 headgroups into the strong, unassigned density in the $E2.P_i^{SPM}$ data. The PC headgroup fits the best among the tested head groups, but due to low resolution, it cannot be excluded that the density corresponds to other lipids, ligands and/or glycosylation.

Supplementary Figure 11.

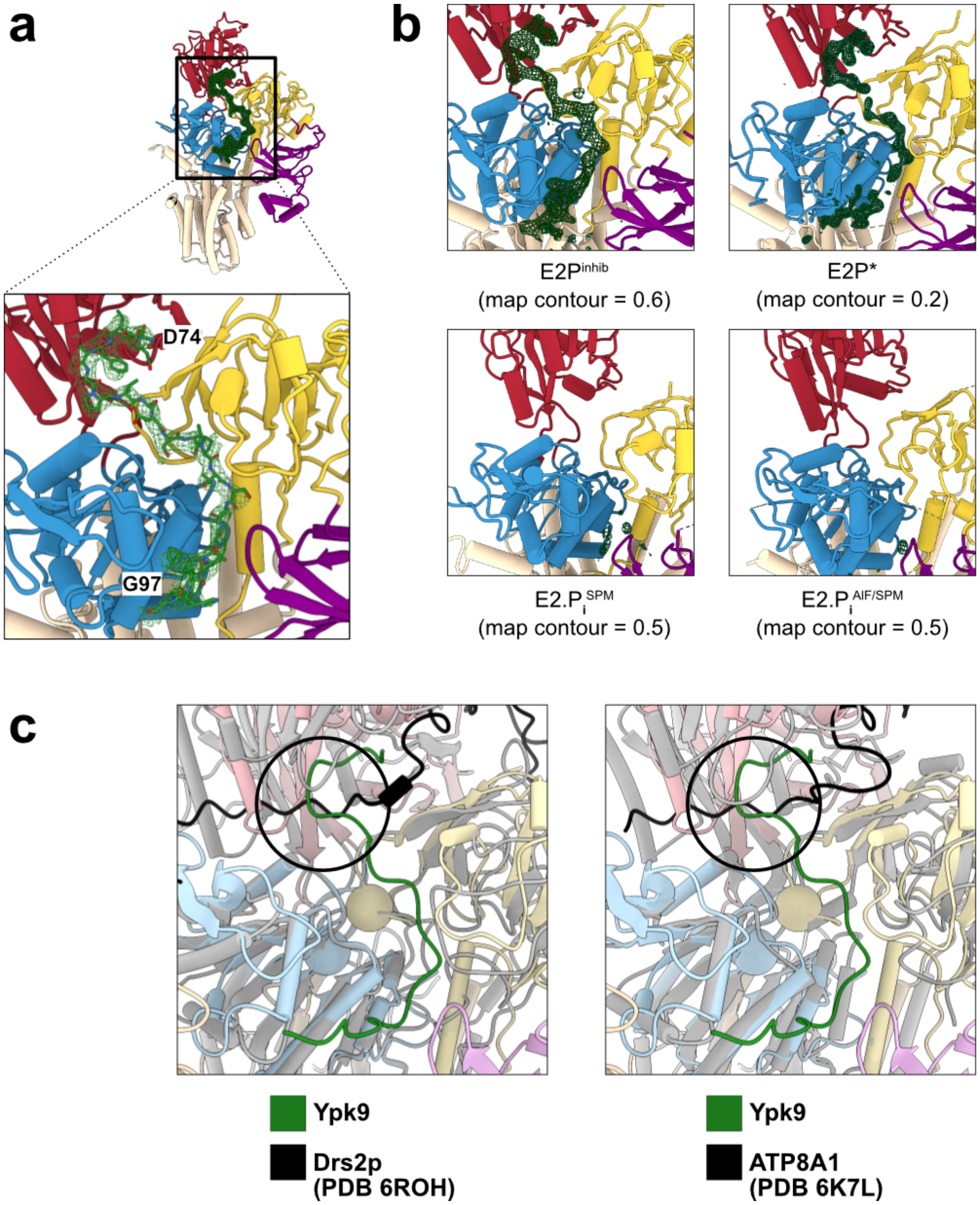

Supplementary Figure 11. Autoinhibition. a EM density corresponding to the N-terminal autoinhibition loop in the E2P^{inhib} state is shown in green mesh. **b** While strong density is present in the E2P^{inhib} map, it is much weaker in the E2P* state, and absent in the E2.P$_i$ conformations. **c** Structural alignment of Ypk9 in the E2P^{inhib} state (colored in blue, red, yellow, purple and green) to the P-domains of similar states of the P4-ATPases Drs2p and ATP8A1 (grey and black). The Ypk9 autoinhibition loop overlaps with the autoinhibitory domain of both P4-ATPases around the nucleotide binding site.

Supplementary Table 3. Primers for cloning of CtYpk9.

Primers(Forward/Reverse)	
ctP5B-22b-F	TCTTTATTTTCAGGGCATGGACGGATCGCGGGGTACAGCCCCT
ctP5B-22b-R	TTAGCAGCCGGATCTCATCAGAAAACTAATTTCTCCTTCACAATCTTG
Δintron1-F	CATGATGAGCTATTTGCTGGCCCCGTG
Δintron1-R	AGCAAATAGCCTCATCATGTGCCATTTCAACTCCCT
Δintron2-F	GTCAGTGAGATCCTTACGCCTTTTTATGC
Δintron2-R	CGTAAGGATCTCACTGACAAGGAGTTGAAG
Δintron3-F	ACCAAGGAGACAAGGAGGCGTCTTCGTG
Δintron3-R	CTCCTTGTCTCCTTGGTCTCCAGGAGACTTG
Δintron4-F	GTGGATTCTGGCGGACTTTCCCATCTAGC
Δintron4-R	AAGTCCGCCAGAATCCACCCCTGAAAACC
Δintron5-F	TCGGAGAGGCACCTCCAGCGGTCG
Δintron5-R	TGGAGGTGCCTCTCCGATGCTCATCTCTTGGGA
Δintron6-F	GGAAACATTGCCCTCCGACTTCGACGAG
Δintron6-R	GGAGGGCAATGTTTCCGGCCTGCAGATCTC
Δintron7-F	GAAACCTGGCCATTCCTATCTGCCGTTGC
Δintron7-R	TAGGAATGGCCAGGTTTCCAGCGGCGACTG
ΔN100-F	CAATTCTAAGATAATTATGTCT GTG GCC AGC TTT TCT TTC
ctP5B-C-GFP-F	CAATTCTAAGATAATTATGGACGGATCGCGGGGTAC
ctP5B-C-GFP-R	ATTGAAAATACAAATTTTCGAAAACTAATTTCTCCTTCACAATCTTGT AC
ctP5B-N-GFP-F	TTG TAT TTT CAA TCT ATG GACGGATCGCGGGGTAC
ctP5B-N-GFP-R	TTGATATTGGATCATCTA TCAGAAAACTAATTTCTCCTTCACAATC

Supplementary Figure 12.

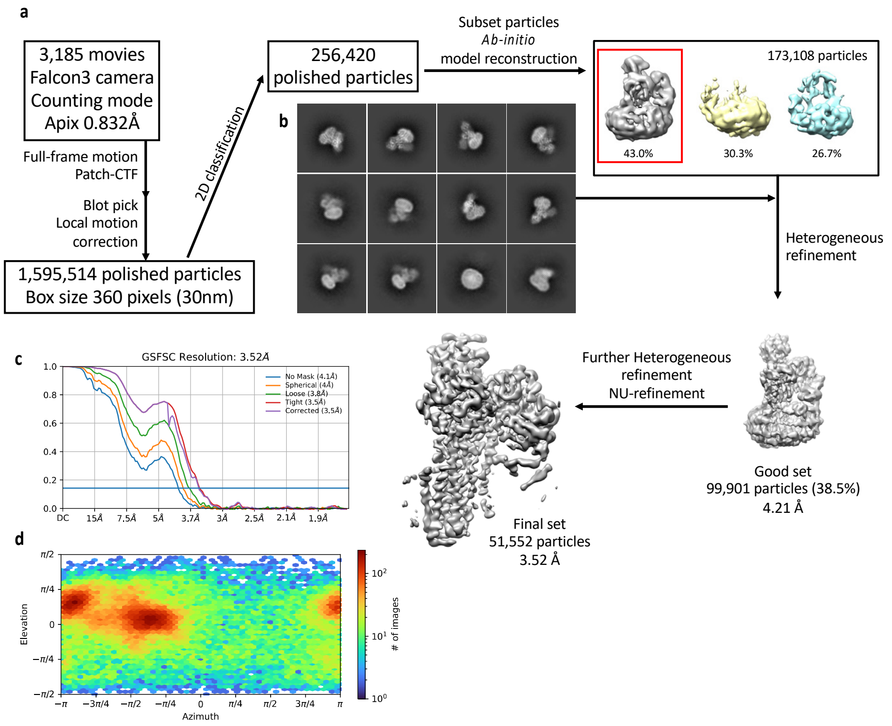

Supplementary Figure 12. Data processing of the E2P^{inhib} state. a Data processing flow-chart. Please see the Methods section and Table S1 for details. **b** Representative 2D class averages. The box size was 30 nm. **c** Gold standard Fourier shell correlation (FSC) curve of the final map. **d** Particle orientation distributions in the final 3D reconstruction.

Supplementary Figure 13.

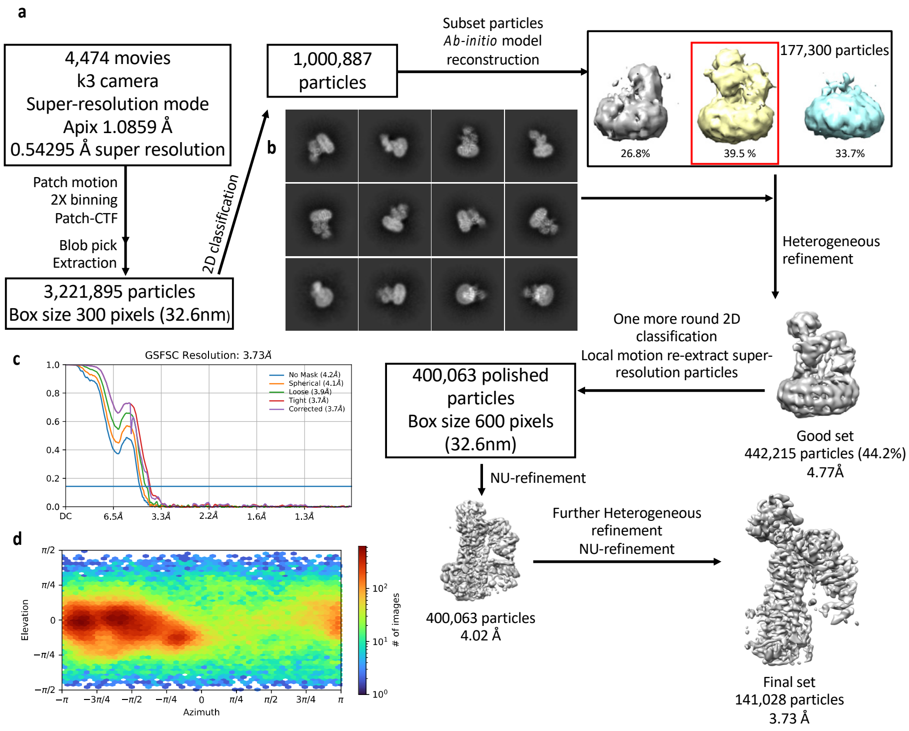

Supplementary Figure 13. Data processing of the E2P* state. a Data processing flow-chart. Please see the Methods section and Table S1 for details. **b** Representative 2D class averages. The box size was 30 nm. **c** Gold standard Fourier shell correlation (FSC) curve of the final map. **d** Particle orientation distributions in the final 3D reconstruction.

Supplementary Figure 14.

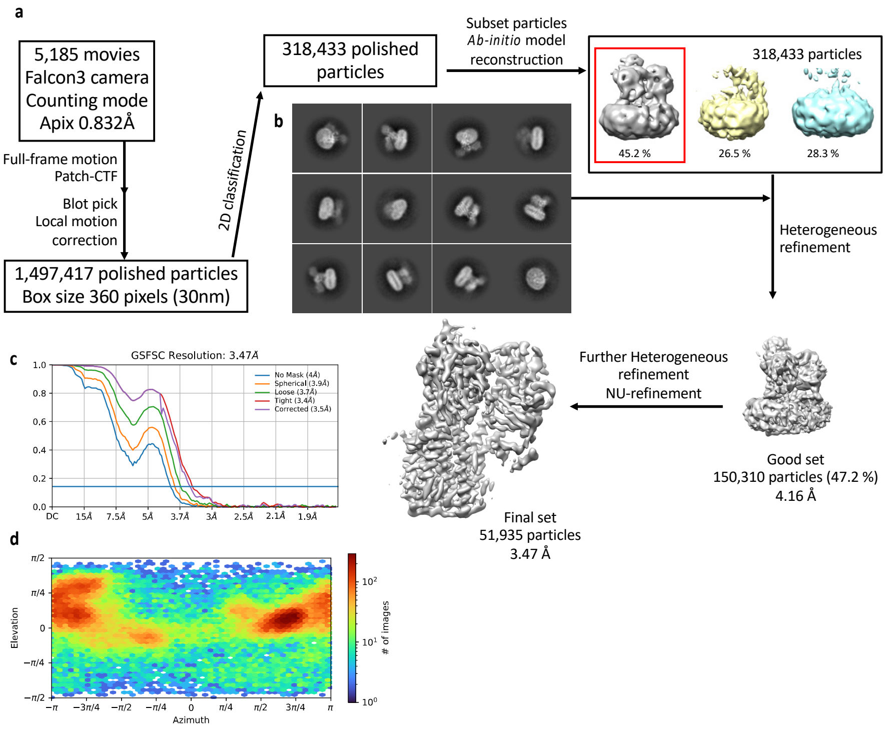

Supplementary Figure 14. Data processing of the E2.P$_i^{SPM}$ state. a Data processing flow-chart. Please see the Methods section and Table S1 for details. **b** Representative 2D class averages. The box size was 30 nm. **c** Gold standard Fourier shell correlation (FSC) curve of the final map. **d** Particle orientation distributions in the final 3D reconstruction.

Supplementary Figure 15.

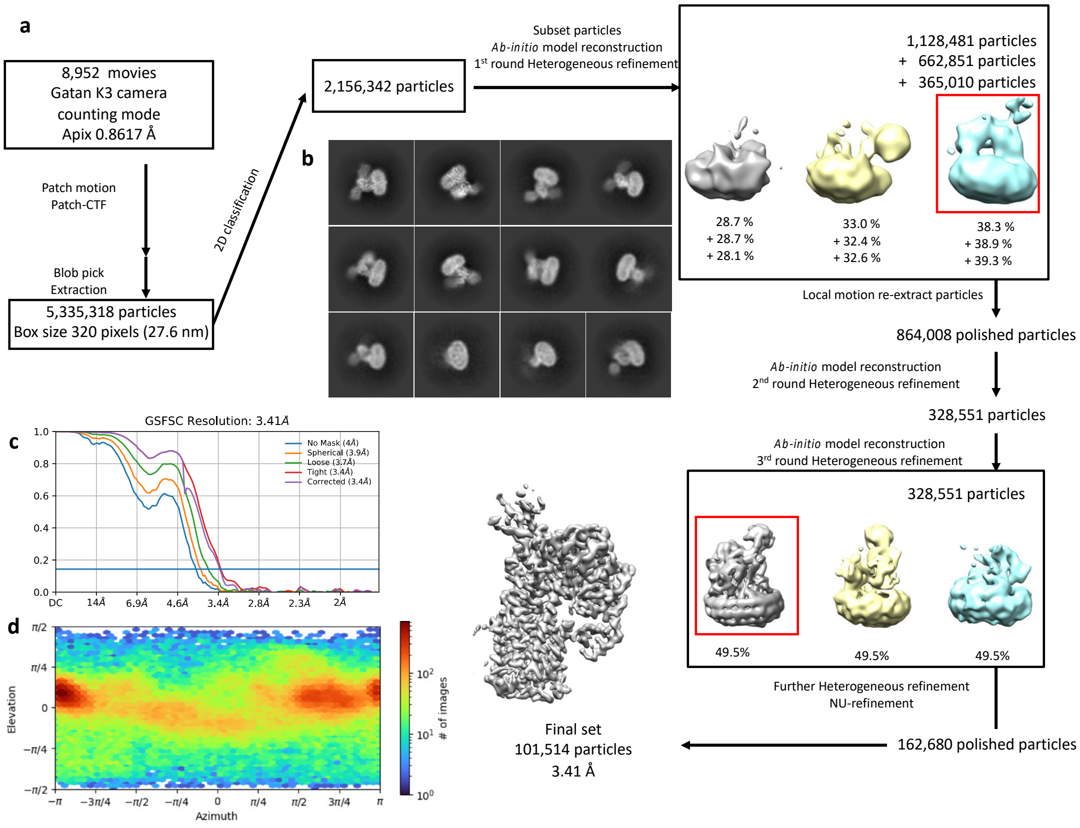

Supplementary Figure 15. Data processing of the E2.P$_i^{AlF/SPM}$ state. a Data processing flow-chart. Please refer to Experimental Procedures and Table S1 for details. **b** Representative 2D class averages. The box size is 30 nm. **c** Gold standard Fourier shell correlation (FSC) curve of the final map. **d** Particle orientation distributions in the final 3D reconstruction.
