# Supplementary material for: Structure and transport mechanism of P5B-ATPases
Source: Nat Commun. 2021 Jun 25;12:3973. doi: 10.1038/s41467-021-24148-y (PMC8233418; doi:10.1038/s41467-021-24148-y)
Supplement: Supplementary file 3 — Reporting Summary [file 41467_2021_24148_MOESM3_ESM.pdf]

nature research

Corresponding author(s): Pontus Gourdon

Last updated by author(s): May 26, 2021

Reporting Summary

Nature Research wishes to improve the reproducibility of the work that we publish. This form provides structure for consistency and transparency in reporting. For further information on Nature Research policies, see our Editorial Policies and the Editorial Policy Checklist.

Statistics

For all statistical analyses, confirm that the following items are present in the figure legend, table legend, main text, or Methods section.

n/a	Confirmed	
☐	☒	The exact sample size (*n*) for each experimental group/condition, given as a discrete number and unit of measurement
☐	☒	A statement on whether measurements were taken from distinct samples or whether the same sample was measured repeatedly
☒	☐	The statistical test(s) used AND whether they are one- or two-sided<br>*Only common tests should be described solely by name; describe more complex techniques in the Methods section.*
☒	☐	A description of all covariates tested
☒	☐	A description of any assumptions or corrections, such as tests of normality and adjustment for multiple comparisons
☒	☐	A full description of the statistical parameters including central tendency (e.g. means) or other basic estimates (e.g. regression coefficient) AND variation (e.g. standard deviation) or associated estimates of uncertainty (e.g. confidence intervals)
☒	☐	For null hypothesis testing, the test statistic (e.g. *F*, *t*, *r*) with confidence intervals, effect sizes, degrees of freedom and *P* value noted<br>*Give P values as exact values whenever suitable.*
☒	☐	For Bayesian analysis, information on the choice of priors and Markov chain Monte Carlo settings
☒	☐	For hierarchical and complex designs, identification of the appropriate level for tests and full reporting of outcomes
☒	☐	Estimates of effect sizes (e.g. Cohen's *d*, Pearson's *r*), indicating how they were calculated

Our web collection on statistics for biologists contains articles on many of the points above.

Software and code

Policy information about availability of computer code

Data collection	Single particle cryo-EM data were collected on Titan Krios electron microscopes with a Falcon3 detector or a Gatan K3 detector in super-resolution mode
Data analysis	Cryo-EM data were processed with cryosparc v 2.15.0(https://cryosparc.com). E2P* and E2.PiAlF/SPM model building was performed using USCF chimera 1.14, Wincoot 0.9.2 , and phenix1.18.2. E2Pinhib and E2.PiSPM model building was performed by using coot 0.9.4.1 , and phenix1.19. The functional data was assessed using Graph Pad Prism 9. Figures were generated using UCSF Chimera-1.15, and ChimeraX version 1.1 (2020-10-07). Alignments were performed using Clustal Omega (online), and visualized using ESPript 3.0 44

For manuscripts utilizing custom algorithms or software that are central to the research but not yet described in published literature, software must be made available to editors and reviewers. We strongly encourage code deposition in a community repository (e.g. GitHub). See the Nature Research guidelines for submitting code & software for further information.

Data

Policy information about availability of data

All manuscripts must include a data availability statement. This statement should provide the following information, where applicable:

- Accession codes, unique identifiers, or web links for publicly available datasets
- A list of figures that have associated raw data
- A description of any restrictions on data availability

The following accession codes and identifiers were used in the work:
The amino acid sequence: uniprot G0S7G9.
The structures and Cryo-EM maps have been deposited to the PDB with the following accession numbers: PDB-ID 7OP8/EMD13014-for E2Pinhib, PDB-ID 7OP5/EMD-13013 for E2P*, PDB-ID 7OP1/EMD-13011 for E2.PiAlF/SPM and PDB-ID 7OP3/EMD-13012 for E2.PiSPM. The raw data of the functional assay raw is provided

in the source data file.

Field-specific reporting

Please select the one below that is the best fit for your research. If you are not sure, read the appropriate sections before making your selection.

[x] Life sciences [ ] Behavioural & social sciences [ ] Ecological, evolutionary & environmental sciences

For a reference copy of the document with all sections, see nature.com/documents/nr-reporting-summary-flat.pdf

Life sciences study design

All studies must disclose on these points even when the disclosure is negative.

Sample size	Complete Cryo-EM statistics are provided in the Methods section, Supplementary Table 1 and Supplementary Figures 12-15. Sample size determination was not used for the enzymatic assay.
Data exclusions	Complete Cryo-EM statistics are provided in the Methods section, Supplementary Table 1 and Supplementary Figures 12-15.
Replication	The two full-length protein forms were produced and and purified multiple times. Multiple Cryo-EM grids were prepared and frozen, data was collected for one selected grid for each state. The activity assay was performed by three independent measurements, and all data are included in the analysis (no failed attempts).
Randomization	Extracted particles were randomly assigned to calculate gold-standard FSC.
Blinding	No blinding was applied as no group allocation was used.

Reporting for specific materials, systems and methods

We require information from authors about some types of materials, experimental systems and methods used in many studies. Here, indicate whether each material, system or method listed is relevant to your study. If you are not sure if a list item applies to your research, read the appropriate section before selecting a response.

Materials & experimental systems

n/a	Involved in the study
[x]	[ ] Antibodies
[ ]	[x] Eukaryotic cell lines
[x]	[ ] Palaeontology and archaeology
[x]	[ ] Animals and other organisms
[x]	[ ] Human research participants
[x]	[ ] Clinical data
[x]	[ ] Dual use research of concern

Methods

n/a	Involved in the study
[x]	[ ] ChIP-seq
[x]	[ ] Flow cytometry
[x]	[ ] MRI-based neuroimaging

Eukaryotic cell lines

Policy information about cell lines

Cell line source(s)	PAP1500 S. cerevisiae strain was used for the protein production
Authentication	Not authenticated
Mycoplasma contamination	no
Commonly misidentified lines (See ICLAC register)	n/a
